# Assessment of the knowledge, attitudes, and practices toward human tuberculosis amongst rural communities in Chad

**DOI:** 10.3389/fvets.2024.1334303

**Published:** 2024-05-16

**Authors:** Lamireou Didi, Mahamat Fayiz Abakar, Ngandolo Bongo Naré Richard, Adou Djané, Hamit Kessely, Yaya Issaka, Serge Diagbouba, Belem Adrien Marie Gaston, Jakob Zinsstag, Bassirou Bonfoh, Salome Dürr

**Affiliations:** ^1^Institut de Recherche en Elevage Pour le Développement, N'Djamena, Chad; ^2^Centre Suisse de Recherches Scientifiques (CSRS), Adiopodoume, Côte d'Ivoire; ^3^Institut National de Santé Publique (INSP) Côte d'Ivoire, Abidjan, Côte d'Ivoire; ^4^Centre de Support en Santé Internationale–Programme d'Appui au District Sanitaires au Tchad, N'Djamena, Chad; ^5^Université Nazi Boni, Ecole Doctorale Sciences et Agronomie: Biologie Appliquée et Modélisation des Systèmes Biologiques, Bobo Dioulasso, Burkina Faso; ^6^Swiss Tropical and Public Health Institute, Basel, Switzerland; ^7^Veterinary and Public Health Institute, Faculty of Vetsuisse, University of Bern, Bern, Switzerland

**Keywords:** tuberculosis, KAP, disease awareness, rural population, Chad, surveillance, prevention

## Abstract

**Introduction:**

Tuberculosis (TB) is an infectious zoonotic disease caused by bacteria belonging to the *Mycobacterium tuberculosis* complex. In sub-Saharan African countries such as Chad, TB is endemic and causes a high burden on humans and animals through morbidity, mortality, and reduced productivity in livestock. To effectively prevent and control the disease, strong coordination between policymakers, health and veterinary services, civil society organizations, and communities is needed. It also requires an understanding of the knowledge the communities have regarding TB. However, such knowledge is under-investigated, especially in rural areas. How knowledge affects people's attitudes and practices is also unclear. The main objective of this study was to investigate the knowledge, attitudes, and practices (KAP) of Chadian rural communities to better involve them in TB surveillance programs.

**Methods:**

A survey was conducted in 2021 in five rural health centers. Face-to-face interviews were conducted with persons suspected of having TB, and data on KAP were recorded and analyzed.

**Results:**

In total, 139 participants were enrolled. Overall, the knowledge and attitude of the participants were found to be good to moderate, with 126 (90.6%) and 97 (69.7%) having good knowledge and attitude, respectively. However, their practices were found to be rather weak, with only 40 (28.7%) participants having good practices. Men were found to have good knowledge about the disease significantly more often than women. Poor attitude was significantly associated with a mobile lifestyle compared to settled lifestyles and with farmers (mainly engaged in agriculture) compared to breeders (livestock keepers). Poor health practices were associated more with men than women and with settled lifestyles compared to a mobile lifestyle. Good practices were found to be in line with good knowledge and good attitudes; however, in the analyses, the association was not significant [OR knowledge = 5.83 (95% C.I. 0.6842.83), *p* = 0.112; OR attitude = 2.09 (95% C.I. 0.875.04), *p* = 0.100]. Furthermore, attitude was not associated with knowledge [OR = 1.03 (95% C.I. 0.303.55), *p* = 0.964].

**Discussion and conclusion:**

Our study highlights the need for targeted sensitization and awareness campaigns for communities with poor knowledge and attitudes regarding TB. These campaigns should also include practical training to increase the level of good practice rather than simply providing knowledge.

## 1 Introduction

Mammalian tuberculosis is a chronic bacterial disease in mammalians, including humans, caused by members of the *Mycobacterium tuberculosis* complex (MTBC) (1). The main members of the MTBC include *M. tuberculosis, M. bovis*, and *M. caprae*, amongst others. Mammalian tuberculosis (TB) is globally distributed. The disease resulting from MTBC infection causes economic losses in livestock due to deaths, lost productivity, and trade restrictions. In addition, it continues to be a public health concern in many countries, with people getting infected by drinking unpasteurized or uncooked milk. The zoonotic nature of TB is thus particularly prevalent in countries without control programs for cattle and without extensive milk pasteurization.

In 2019, ~10 million persons were infected by TB worldwide, leading to 1.2 million deaths (2). In 2020, COVID-19 negatively impacted global progress in reducing tuberculosis, resulting in reduced surveillance and a significant drop in the number of people newly diagnosed and reported with TB compared to 2019 (2). In addition, drug-resistant TB is emerging as a significant challenge to the effective treatment and disease eradication efforts. Anti-microbial resistance is further estimated to push an additional 28.3 million people into extreme poverty by 2050 due to the high costs of treatment and chronic infections (3).

Africa accounted for 25% of the 10 million global human TB cases reported in 2020 (2). In 2014 and 2015, all member states of the World Health Organization and the United Nations committed to reducing TB infections by 2030 and to eliminating the disease in humans by 2050 based on the following three pillars:

1. Integrated, people-centered care and prevention aimed at early and universal access to diagnosis and treatment of all forms of TB.
2. Strong policies and supportive systems aimed at enforcing government leadership, civil society, and private sector engagement, as well as universal health coverage, social protection, poverty alleviation, and action on the social determinants of TB.
3. Intensified research and innovation to accelerate discovery, development, and rapid uptake of new tools, interventions, and strategies with specific indicators, milestones, and targets for 2020, 2025, 2030, and 2035.

The second pillar calls for a strong coalition and coordination between policymakers and health services, with a significant role for civil society organizations and communities (4). This approach is not implemented effectively in the health systems of many sub-Saharan African countries, including Chad (2).

Chad is located in the Sahel region of Africa, with an estimated population of 16 million people (5). It is a pastoral country with a large cattle population, estimated at 114 million heads (6). The income from pastoral and agricultural activities supports ~40% of the national economy. Tuberculosis is a major disease in Chad, with an incidence of 144 cases/100,000 inhabitants in 2020 (7) of zoonotic infections reported at the national level (8). Although the disease is increasingly suspected and diagnosed in health centers and abattoirs in the country (9, 10), the underestimation of TB cases is still a large challenge. For instance, going by the incidence rate reported above, 24,919 human TB cases should have been identified in 2021. However, only 13,432 cases were reported to the national TB surveillance system for 2021 (7), indicating that TB may be underreported in Chad.

The incidence of zoonotic TB cases is higher in rural than urban areas, with 1.4% of all human cases in the African population (11), where people are highly exposed due to close contact with their livestock, facilitating transmission of zoonotic diseases (12). In addition, these populations are often hard to reach by public services, including health and education (11–13). Consequently, they have poor access to health services, leading to non-existent or inadequate treatment and eventually death. Most patients diagnosed with TB at health centers in rural areas in Chad are already at the infectious stage of the disease, with often unknown duration of exposure of their family members before seeking medical attention from available health services (8). TB is generally a treatable condition, and the clinical stage of the disease and its transmission to others can be avoided. The infection can be cured when appropriate measures are taken early by the exposed population. However, this requires timely detection of the infection.

One of the main challenges for effective TB surveillance and control in rural populations is to establish collaboration between the communities, medical health services, and policymakers (7). Community members constitute the first step of the reporting chain and are keys to a prompt reaction to suspected TB cases. Therefore, knowledge about their understanding and perception of the disease is crucial to improve reporting and treatment systems. The aim of the present study was to assess the knowledge, attitudes, and practices (KAP) of the rural community population regarding TB infection, transmission, and sources of information in two health districts in Chad. This will serve to understand the target population's perception of TB infection, prevention, and control and will help to develop sensitization campaigns.

## 2 Materials and methods

### 2.1 Study site and study design

#### 2.1.1 Study site

**Figure 1:**
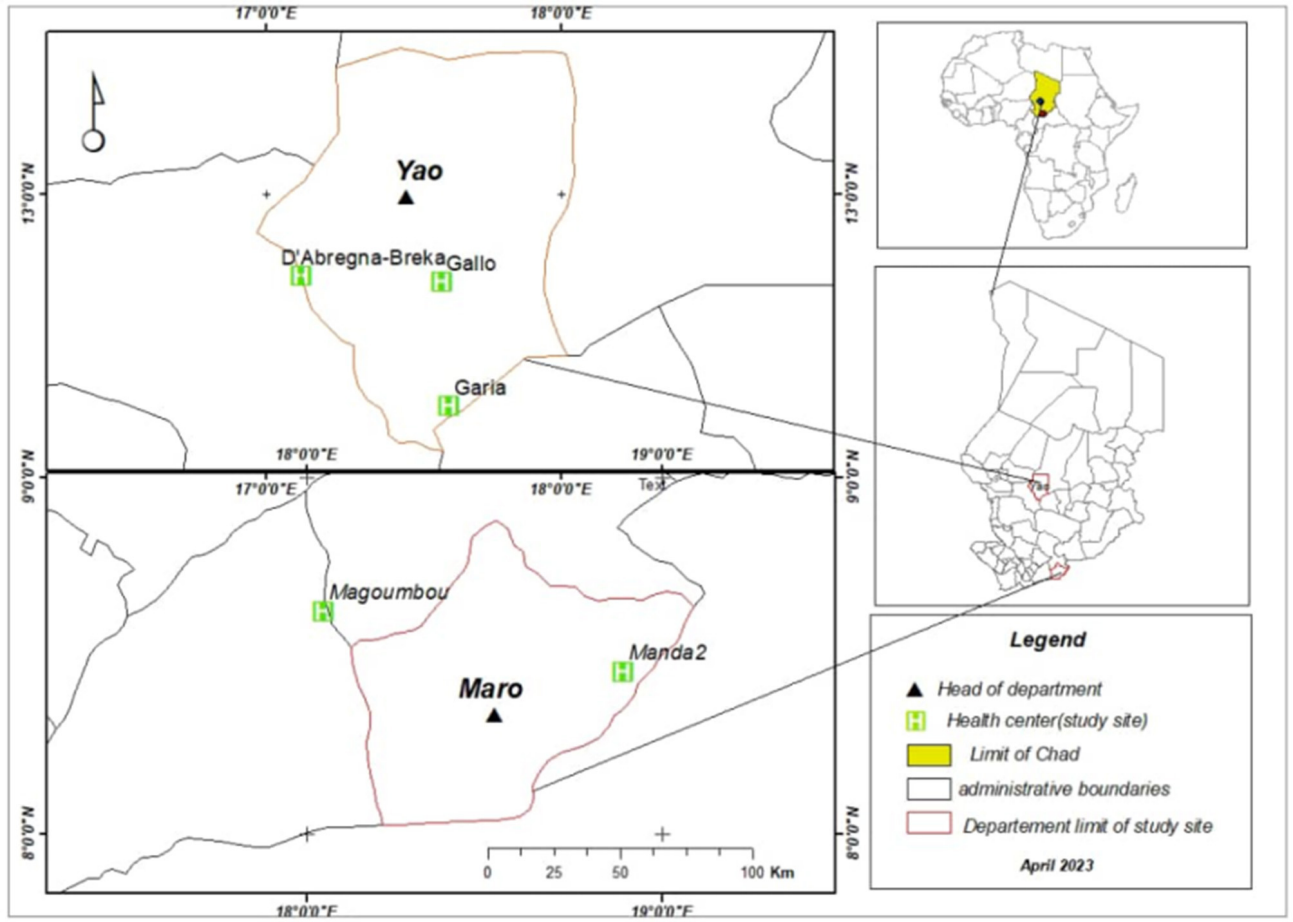
Districts and sites of the five health centers of the knowledge, attitude, and practice survey conducted in 2021 in Chad.

The study was conducted in two health districts of Chad: Yao in the Batha province in the center of the country and Danamadji in the Moyen Chari province in the southeast, bordering the Central African Republic (Figure 1). These provinces are known to be areas of intense farming, where programs and research on livestock and community health are prevalent. These two particular health districts were chosen because of existing collaboration with health centers in these regions. In the Yao health district, three health centers were included, namely Galo, Garia, and Abregna-Breka, whereas in the Danamadji health district, two health centers were included—Manda2 and Magoumbou.

#### 2.1.2 Study design and study population

These rural provinces include both sedentary farmers and mobile pastoralists, who live side by side and form the study population. While both farmers are cattle and small ruminant breeders, the settled farmers also cultivate crops (14). The ethnic group in Batha province mainly consists of Arabs (mobile population) and Fulani (settled and mobile population). In contrast, in Moyen Chari province, Arabs (mobile population), Fulani (mobile population), and Sara (settled population) are predominant. While farmers are engaged in agriculture for food and growing cereals and vegetables, breeders keep and breed cattle, goats, or sheep.

Both regions have a high cattle density. Mobile pastoralists in these provinces have a transhumance lifestyle based on seasonal movement. Their transhumance routes depend on the availability of pasture for their livestock.

Our study was conducted from September to November 2021, during a period of the year when the mobile pastoralists were present close to the villages. This is also the period when they frequent the national public health structures, including the health centers. This period also corresponds with agricultural product harvesting, during which time settled farmers are also present in their areas to sell harvest products. Consequently, health center attendance is usually high during this period, increasing the chance of recruiting a significant number of participants from both settled farmers and mobile pastoralists.

Our study was undertaken with persons suspected of having TB who came to the health centers for diagnosis or treatment. Individuals were included in the study if they were at least 16 years old, presented with fever and cough for more than 2 weeks, and gave their full informed consent to participate in the study. Many people visit health centers for different reasons. When they suspected TB symptoms, such as cough and fever, the respective health center selected them for the study and performed the interviews. We aimed to interview an equal distribution of patients between health centers, and according to the census in Chad, populations are nearly the same in both provinces (Batha: 529,546 residents, Moyen Chari: 571,639 residents). The interviews lasted between 5 and 10 min. Confidentiality of collected data from the individual interviews was guaranteed by storing the collected data in a locked and secured database and using coded information that cannot be traced back to the name of the individuals.

#### 2.1.3 Data collection

The objective of the study was explained to the persons suspected of having TB, and their consent was requested for participation in the study. After the agreement, a face-to-face interview was conducted.

The questionnaire consisted of four parts:

- The first section contained 19 questions on the demographic characteristics (gender, age, occupation, ethnic group of the participant), the occurrence of general symptoms (weakness, weight loss, fever, loss of appetite, tiredness, night sweats), and specifics symptoms (cough, hemoptysis, thoracic pain, presence of ganglion) on tuberculosis.
- The second part included 10 questions concerning the evaluation of the participant's knowledge of TB infection and treatment (knowledge about the disease, channel of information, TB transmission pathways, and zoonotic characteristics of the disease).
- The third part contained six questions concerning the participant's attitudes toward seeking healthcare at health centers or hospitals. These questions were related to aspects that we asked the respondents about, such as what they believed was right or wrong or should be done or not done. We also asked whether sick people were excluded from their communities.
- The fourth part had eight questions about practices related to TB prevention (e.g., consumption of untreated milk or meat, protection while handling sick animals, adherence to medical instructions for TB treatment, etc.) and about what they actually do. These questions included commonly known practices that need to be followed. But it was of interest to investigate whether these were practically possible to undertake.

The full questionnaire, translated into English, is attached as Supplementary material. The questionnaire was written in French. During the interview, local Arabic, French, or other local languages spoken in the surrounding villages were used as communication languages. The data was collected on paper.

### 2.2 Data management and statistical analysis

The collected data were entered twice into Access software to identify data entrance errors and then transferred to Microsoft Excel and RStudio version 4.2.2 software for descriptive and analytical statistical analysis.

The levels of knowledge, attitude, and practice were classified in binary codes for each participant, according to their responses. For knowledge, two levels were defined. When participants correctly answered six or more questions out of the ten questions, it was considered as good knowledge; five or fewer correct answers were considered as poor knowledge. For attitude, two levels were defined, with four or more correct answers out of the six questions considered as good attitude and three or fewer correct answers considered as poor attitude. For practices, the two levels were defined with five or more suitable answers out of the eight questions considered as good practices and four or fewer correct responses considered as poor practices.

The proportions of the levels of knowledge, attitudes, and practices were calculated and presented overall and stratified by gender, age, lifestyle (settled vs. mobile), occupation, and health center. Univariable logistic regression models were performed to investigate the associations between the level of knowledge, attitude, and practice, respectively, with the explanatory variables being gender (male vs. female), age (as a continuous variable), lifestyle, and occupation. The occupation variable was categorized into housewives, farmers, breeders, laborer, and students. We also explored whether the outcome variables were associated with the health centers by using univariable logistic regression models. Finally, we investigated the association between the three outcome variables—knowledge, attitude, and practice—using logistic regression models. Statistical significance was set with a *p*-value of 0.05 with a confidence level of 95%. The database is included in the Supplementary material.

### 2.3 Ethics statement

Ethical clearance was obtained from the Chadian Bioethics Committee (N°0200/PR/MESRI/DG/CNBT/2020 of November 6, 2020). The Ministry of Public Health and the Ministry of Livestock approved the study by providing a permit to access health centers. Each participant signed an informed written consent form before data collection.

## 3 Results

### 3.1 Study population

In total, 139 persons suspected of having TB who visited the health centers for TB diagnosis and treatment were enrolled in the survey. The health centers were well-represented, and men were included slightly more than women. The age of the participants ranged from 17 to 74 years, with a median age of 40 years. Most participants were from the age group ranging from 31 to 45 years. In terms of occupation, most of the study participants were housewives and farmers. Most were from settled populations, with a minority from mobile pastoralists, who overall account for a lower proportion of the population in Chad. Eighty (57.5%) participants presented at least two specific TB symptoms (cough, hemoptysis, thoracic pain, or presence of ganglion), and 48 (34.5%) had at least three general non-specific symptoms (weakness, weight loss, fever, or loss of appetite).

### 3.2 Knowledge, attitude, and practices

#### 3.2.1 Knowledge

**Figure 2:**
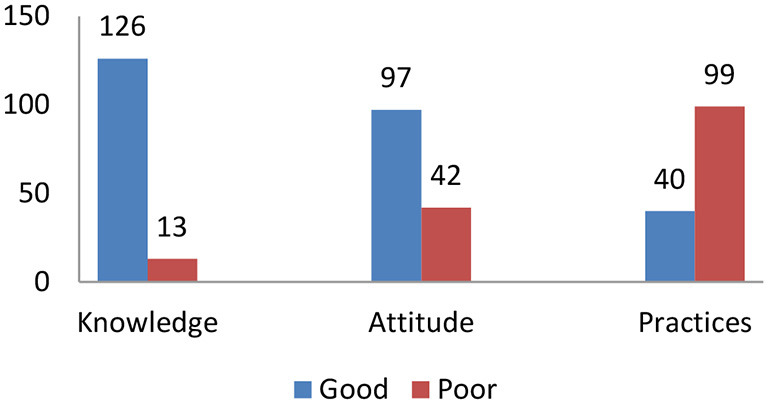
Absolute numbers of knowledge, attitude, and practice levels toward TB of 139 participants in the survey in rural Chad, 2021.

Out of the 139 participants, 125 (89.9%) affirmed that they had been informed about human TB, and 98 (70.5%) about animal TB. Fourteen participants (10.1%) indicated that they did not know about TB. The main source of information on human and animal TB was through radio (*n* = 55, 39.5%) and during meetings with family (44, 31.6%), while place of work (5, 3.5%), school (4, 2.8%), mosque (2, 1.4%) and television (1, 0.7%), contributed less as an information source. Overall, 101 participants (72.7%) recognized TB as contagious, and 135(97.1%) said that TB is a lethal disease. A total of 54 (38.8%) participants stated they knew the cause of the disease, while the remaining 85 (61.1%) were unaware. In addition, 124 participants (89.2%) were aware that TB can be transmitted between humans and animals, while 15 (10.7%) did not believe in transmission. Concerning TB transmission mode between humans and animals and vice-versa, 97 participants (69.7%) specified inhalation as the only transmission pathway to humans, while 42 (30.2%) reported ingestion of contaminated products as a potential zoonotic transmission route. In terms of medical treatment, 130 (93.5%) participants stated that tuberculosis can be cured. Overall, 126 (90.6%) participants were able to correctly answer five or more questions regarding TB knowledge and were thus judged to have a good level of knowledge according to the criteria laid out. In contrast, 13 (9.3%) had a poor level of TB knowledge (Figure 2, Table 1).

**Table 1:** Distribution of good and poor knowledge, attitude, and practice among participants stratified by gender, age, lifestyle, occupation, and health center.

**Variables**	**Number**	**Knowledge**		**Attitude**		**Practice**	
	**Total: 139 (100%)**	**Good (*****n** =* **126, 90.6%)**	**Poor (*****n** =* **13, 9.3%)**	**Good (*****n** =* **97, 69.8%)**	**Poor (*****n** =* **42, 30.2%)**	**Good (*****n** =* **40, 28.8%)**	**Poor (*****n** =* **99, 71.2%)**
**Gender**							
Male	79 (57%)	75 (94.9%)	4 (5.1%)	55 (69.6%)	24(30.3%)	17 (21.5%)	62 (78.4%)
Female	60 (43.1%)	51 (85%)	9 (15%)	42(70%)	17 (28.3%)	23 (38.3%)	37 (62%)
**Age (years)**							
17–30	43 (30.9%)	41 (95.3%)	2(4.6%)	40 (93%)	3 (7%)	17 (39.5%)	26 (60.4%)
31–45	48 (34.5%)	46 (96%)	2 (4.1%)	44 (92%)	4 (8.3%)	15 (31.2%)	33 (68.7%)
46–∞	48 (34.5%)	41 (85.4%)	7 (14.5%)	42 (87.5%)	6 (12.5%)	9(18.7%)	39(81.3%)
**Life style**							
Settled	116 (83.4%)	107 (92.2%)	9 (7.8%)	108 (93.1%)	8 (6.9%)	30 (25.9%)	86 (74.1%)
Mobile	23 (16.5%)	21 (91.3%)	2 (8.7%)	18 (78.2%)	5 (21.8%)	11 (47.8%)	12 (52.2%)
**Occupation**							
Student	4 (2.9%)	4 (100%)	0	3 (75%)	1 (25%)	3 (75%)	1 (25%)
Farmer	42 (30.2%)	39 (93%)	3 (7.1%)	33 (78.5%)	9 (21.4%)	9 (21.4%)	33 (78.5%)
Housewife	57 (41%)	49 (86%)	8 (14%)	40 (70%)	17 (30%)	21 (37%)	36 (63.1%)
Laborer	18 (13%)	17 (94.4%)	1 (5.5%)	12 (67%)	6 (33.3%)	2 (11.1%)	16 (88.9%)
Breeder	18 (13%)	17 (94.4%)	1 (5.5%)	9 (50.0%)	9 (50.0%)	5 (27.8%)	13 (72.2%)
**Health center**							
Galo	28 (20.1%)	25 (89.2%)	3 (11%)	24 (86%)	4 (14.2%)	3 (10.2%)	25 (89.2%)
Garia	30 (21.5%)	29 (97%)	1 (3.3%)	24 (80%)	6 (20%)	0	30 (100%)
AbregnaBreka	28 (20.1%)	28 (100%)	0	27 (96.4%)	1 (4%)	0	28 (100%)
Manda2	23 (16.5%)	16 (69.5%)	7 (30.4%)	21 (91.3%)	2 (9%)	8 (35%)	15 (65.2%)
Magoumbou	30 (21.5%)	30 (100%)	0	30 (100%)	0	30 (100%)	0

#### 3.2.2 Attitudes

In our study, 131 (94.2%) participants said they aim to visit health centers when they are sick, and 8 (5.7%) expressed unwillingness to do so. On treatment, 131 (94.2%) participants believe that modern medicine at the hospital, such as antibiotics, is the best treatment. On the other hand, 8 (5.7%) respondents confirmed that they prefer traditional medicine over that which is offered by modern clinicians. Slightly more than half of the participants (73, 52.5%) stated that it is important to take medication as prescribed, whereas the remaining (66, 47.4%) did not have this opinion. On the other hand, only 57 (41%) participants highlighted the importance of following the doctor's prescriptions, and 82 (58.9%) did not. Similarly, while only 60 (43.1%) participants indicated that it is important to finish their treatment, 79 (56.8%) did not think so. A total of 82 (59.0%) participants reported that those infected with TB are excluded from their communities, while 57 (41%) stated that such an attitude should not be practiced. In summary, 97 (69.7%) participants had good attitudes, and 42 (30.2%) had poor attitudes (Figure 2, Table 1).

#### 3.2.3 Practices

From the participants' responses, we found that 93 (67%) participants sometimes drank raw milk, while 46 (33.1%) did not drink raw milk. In addition, 99 (71.2%) sometimes consumed uncooked meat, while 40 (28.7%) always ate meat cooked. A total of 100 (71.9%) participants reported that they covered their mouths when coughing, but 39 (28.1%) did not do so. While 85 (61.1%) participants generally closed the windows when they were inside, 54 (38.8%) did not close them. When handling animals or biological material during birth, 106 participants (76.2%) did not use protective equipment, while 33 (23.7%) used either gloves and/or a face mask. Also, 32 (23.02%) participants responded that they washed their hands before and after milking, whereas 107 (76.9%) did not wash their hands before or after milking. When animals are sick, 32 (23.02%) respondents reported that they used protective material, such as gloves or face masks, when handling the animals, but 107 (76.9%) did not use protective material. Overall, we classified 40 (28.7%) participants as observing good practices and 99 (71.2%) with poor practices regarding the prevention of TB transmission (Figure 2, Table 1).

#### 3.2.4 Factors associated with knowledge, attitude, and practice

The results of the regression models with demographic factors revealed an association, although not statistically significant by a 5% level, between being male and having better knowledge of TB (Table 2). Being younger was also found to be associated with better knowledge. Poor attitude was significantly associated with having a mobile lifestyle compared to a settled lifestyle and with farmers compared to breeders. Finally, poor practice was found to be significantly associated with men compared to women and was further associated with a settled lifestyle compared to a mobile lifestyle. Overall, settled populations had better attitudes but poorer practices, whereas those having a mobile lifestyle were found to have poorer attitudes but better practices.

**Table 2:** Odds ratios (OR) and corresponding *p*-value of univariable logistic regression model results with knowledge, attitude, and practice of persons suspected of having TB being the outcome variable and the demographic factors being the independent variables, in the survey conducted in rural Chad in 2021.

	**Knowledge**	**Attitude**	**Practice**
	**OR (95% CI)**, ***p*****-value**	**OR (95% CI)**, ***p*****-value**	**OR (95% CI)**, ***p*****-value**
**Gender**			
Female	Reference level	Reference level	Reference level
Male	3.3 (0.96–11.32), 0.057	0.98 (0.47–2.04), 0.961	**0.44 (0.20–0.93), 0.031**
Age	0.97 (0.93–1.01), 0.201	0.99 (0.96–1.01), 0.284	**0.96 (0.93–0.98), 0.008**
**Lifestyle**			
Settled	Reference level	Reference level	Reference level
Mobile	0.62 (0.15–2.48), 0.509	**0.32 (0.13**–**0.80),0.014**	**2.75 (1.09–6.89), 0.031**
**Occupation**			
Breeder	Reference level	Reference level	Reference level
Student	n.a.	0.81 (0.07–8.84), 0.868	**11 (1.01**–**118.87), 0.048**
Farmer	1.30 (0.12–13.49), 0.822	**0.27 (0.08–0.88), 0.031**	1.41 (0.39–5.01), 0.595
Housewife	0.47(0.11–1.89), 0.289	0.64 (0.25–1.62), 0.349	2.13 (0.85–5.32), 0.102
Laborer	1.31(0.12–13.49), 0.822	0.54 (0.16–1.85), 0.332	0.45 (0.08–2.37), 0.352

Significant values are presented in bold.

**Figure 3:**
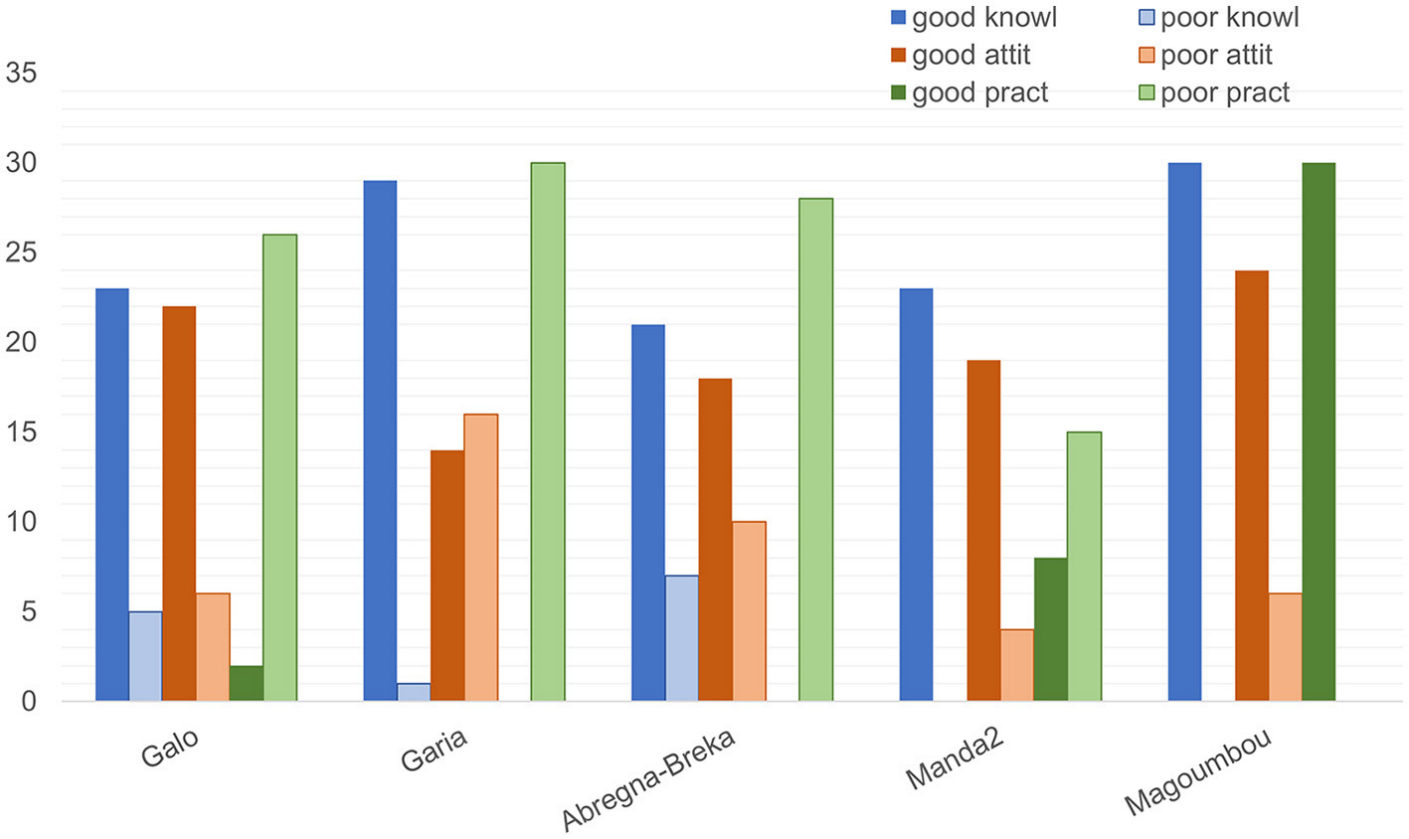
Distribution of knowledge, attitude, and practice among the five health centers included in the survey on TB in rural Chad, 2021.

Good or poor levels of knowledge, attitude, and practice were not evenly distributed between the health centers (Figure 3). Poor knowledge was found to be highest in the health centers of Yao health district (Abregna-Breka, followed by Galo and Garia), whereas no participants recruited from any of the health centers in the Danamadji health district (Manda2 and Magoumbou) showed poor knowledge. Poor levels of attitude were highest in the health centers of Galo and Garia, Manda2, and Abregna-Breka, whereas Magoumbou was the only health center where all recruited participants were found to demonstrate a good attitude. Similarly, all participants from the health center in Magoumbou showed good practice, followed by the health centers of Manda2 and Galo, which also had participants with good practice. Conversely, those from the health centers of Garia and Abregna-Breka were all classified as having poor practices. Due to the large number of zero observations in either good or poor level, the ability to run statistical analysis was limited and thus omitted.

Good practice was found to be in line with good knowledge and a good attitude; yet, for both analyses, the association was not significant [OR knowledge = 5.83 (95% CI 0.68-42.83), *p* = 0.112; OR attitude = 2.09 (95% CI 0.87-5.04), *p* = 0.100]. Attitude was not found to be associated with knowledge [OR = 1.03 (95% CI 0.30-3.55), *p* = 0.964].

## 4 Discussion

In this study, we present the KAP on human TB prevention, transmission, and control of persons suspected of having TB in two rural communities in Chad. Understanding community perceptions and knowledge regarding TB could be important for improving surveillance and control systems, as community engagement is a pivotal element in the implementation of TB programs.

### 4.1 Knowledge assessment

Overall, a large majority (90.6%) of respondents had good knowledge of tuberculosis infection. Similar figures were reported in other studies in South Africa (among rural populations) and Ethiopia (among urban populations), where the majority (94% and 51.6%, respectively) of the study population had good knowledge of TB (15, 16). Nearly all participants in our study had heard about human TB, and almost three-fourths of them knew about animal TB. The latter figure is similar to the findings of a study undertaken in Cameroon, where 81.9% were aware of TB in animals (17).

The main source of information on TB was reported to be the radio, followed by family meetings. The radio was also found to be the main information channel in Somalia, followed by the television (18). This differs from our study, where television played a much less important role in rural areas. This could be explained by the remoteness of the area, where radios are available in each province nowadays, but access to television is still poor. Moreover, family meetings in rural areas constitute a great opportunity for exchanging or sharing news and knowledge, which allows knowledge and information transfer within the family and beyond. Therefore, we suggest that, in collaboration with a national program against TB, key messages related to the disease could be prepared and broadcast in local languages via radio to reach the rural population better. Knowledge transfer during traditional or religious meetings by the chiefs could also be promoted because these channels have also been mentioned in our survey as a source of information.

Although the overall knowledge regarding TB was found to be high in our study, some aspects were known by less than half of the interviewees. We found that 61.1% of participants stated that they did not know the cause of the disease. This is higher than a study conducted in Ethiopia, where 48.7% of participants were found not to know the cause of the disease (19). In addition, knowledge of the zoonotic characteristics of the disease appears to be low, with only one-third of the respondents being aware of the transmissibility of TB between humans and animals. This finding is similar to that reported for Cameroon and Somalia (18, 19). Although people handling animals cannot know whether an animal is infected by TB, there are some symptoms (for example, cough and weight loss) that should alert the keepers to react or protect themselves before handing the animal and requesting etiological diagnostics. Animal keepers should be sensitized to recognize these symptoms and react accordingly. Most participants were aware that inhalation is one of the main transmission modes, as has also been reported by other researchers (20). In Chad, studies evidenced that pulmonary TB caused by *M. bovis* does occur, suggesting that zoonotic transmission from animals to humans happens by inhalation (21). However, transmission by ingestion causing extrapulmonary TB cannot be excluded. The lack of knowledge regarding the cause of TB and its zoonotic transmission is a crucial aspect that needs to be tackled by public awareness, particularly in rural communities where the risk of zoonotic transmission is increased by direct contact between humans and animals or via raw dairy products.

We found that age influences knowledge, with younger age being associated with good knowledge. We believe that this could be due to a higher education level amongst the younger generation in rural communities compared to the older generations. A higher level of education has been shown previously to be related to a higher level of knowledge (22). For example, a study in Ethiopia demonstrated that the level of education influenced disease knowledge (23). In addition, over the past two decades, we noted that there is a new generation of religious leaders who play an important role in education. For example, they encourage community members to visit the health center when they are sick. These leaders may be engaged in disease awareness and knowledge transmission campaigns in a more targeted manner in the future. In contrast to our findings, a study conducted in Nigeria found that knowledge of TB increased with age (24). However, this study was conducted in an urban context, where the education level typically increased with age and where the majority of children had access to schools. This again highlights the importance of education in increasing knowledge and awareness.

In our study, good knowledge was found to be higher in men, although just not statistically significant. This is similar to findings from a study from Vietnam, where men had a higher level of knowledge on TB compared to women (25). This emphasizes the need for education, particularly among women who are often poorly educated in Chad, although they play a crucial role in animal husbandry and disease prevention in rural regions (26). We need to admit that since our study population consisted of persons suspected of having TB, they may have had a better knowledge of TB compared to the rest of the population.

### 4.2 Attitude assessment

Two-thirds of the participants in our study demonstrated good attitudes. This is very similar to a study in North Mecha District, northwest Ethiopia, where 68% had a good attitude (27). The relatively high percentage of good attitudes found in our study may be due to the fact that it was conducted in health centers, thus including only individuals who came to seek healthcare, which itself already constitutes a demonstration of good attitude.

We noted that most of the participants (93.5%) preferred treatment at the hospital rather than traditional medicine-based treatment of TB infection. In Chad, treatment of tuberculosis is provided at hospitals after suspicion is detected in a health center, and those suspected of TB are referred to the hospital for laboratory diagnostics and treatment. This approach is based on the findings from a study conducted in Nigeria, where the majority (80.6%) of respondents preferred treatment at the hospital, and only 1.4% consulted traditional healers (24). Consulting health centers as soon as symptoms appear is particularly important for infectious diseases such as TB to avoid disease transmission to other family members. A critical finding in our study was the high percentage (almost 60%) of TB patients being rejected by the community. The reason for this rejection is not known, and there is a need to sensitize communities to accept and support patients rather than reject them; otherwise, health-seeking practices at health centers will be extremely challenging for patients (28).

Participants living in mobile communities were found to have a poor attitude more often than those living in settled communities. This could be due to the remoteness of mobile pastoralists, which limits their access to health centers. Due to their localization, they require a long time, often up to several days of traveling, to reach a health center, which serves as a demotivating factor for accessing health services. Farmers, compared to breeders, were found more often to have a poor attitude. This could be due to poverty among this group of people since farmers often state they do not have enough money to frequent health centers. This finding could help plan awareness campaigns to improve education among the farming community and change their knowledge and attitude toward more visits to health centers. This is expected to help them prioritize their resources toward health care. As farmers are in the same zones as breeders, an awareness campaign on TB could be carried out by the public health authorities in collaboration with veterinary services.

### 4.3 Practice assessment

Although a large portion of responses relating to attitudes and knowledge, particularly, were classified to be good in our study population, < 30% of participants were found to have a regimen of good practice concerning the prevention and treatment of TB. These poor practices mainly included the consumption of raw milk and meat (67% and 71%, respectively), as well as not using personal protective equipment when handling animals while supporting birthing or during treatment (77%). This is significantly higher than findings from a study in Bangladesh, where only 2.5% of respondents reported drinking raw milk (29), and in Cameroon, where 27% of participants consumed uncooked meat or unpasteurized milk (17). On the other hand, in Somalia, about half of the study population(48.5%) consumed raw milk (18), with a similar observation in Ethiopia, where 52.1% of participants in the study reported consuming raw milk (23). In Kenya, raw milk was reported to be consumed by almost all rural households (99%) surveyed (30). This zoonotic pathway for TB transmission by ingestion of raw milk is a typical threat in rural areas. This is in line with the findings of the knowledge assessment, where we found that a large proportion of participants were not aware of the zoonotic nature of TB. In rural communities, drinking raw milk and eating uncooked meat have been culturally established for generations. Awareness campaigns on the zoonotic character of TB and the health benefits of consuming boiled milk and cooked meat are thus essential for the wellbeing of the population. Knowledge transfer and training on the proper use of personal protective equipment when handling animals during assistance with birth or treatment should also be undertaken. However, simple training and raising awareness may not lead to the full effect of community members using personal protective equipment since, in these remote areas, access to such material is very poor. Therefore, besides awareness campaigns, the government should also provide protective equipment and facilitate communities to easily access it.

We observed poorer practices among men compared to women and an increase in poor practices with age. These observations could be caused by the lack of information on hygiene within these groups in this area. We suppose that women are mostly in charge of milking or cooking food (31), which could explain their better practice.

### 4.4 Influence of the health centers on knowledge, attitude, and practice

We observed that knowledge, attitudes, and practices vary highly among health centers. For all outcome variables, participants from the three health centers located in the Yao health district (Abregna-Breka, Galo, and Garia) were found to have poorer levels of KAP in general, but particularly in practices, than among participants recruited from the two health centers in Danamadji health district (Manda2 and Magoumbou). The reason for this observation is difficult to identify. On the one hand, it may be due to the diversity of the participants recruited in the different health zones, comprising a range of age, occupation, ethnicity, and education level. On the other hand, Yao and Danamadji are located in two different agroecological zones with different lifestyle practices. In Yao, the presence of Lake Fitri attracts more mobile people, who are mostly from the northern part of the country, looking for water. They are generally highly mobile communities that often do not have access to education or any form of awareness against infectious diseases. However, in the southern zone of the country where Danamadji is located, the populations have a more sedentary lifestyle, given the permanent availability of water and cultivable land. Consequently, such populations are often able to access basic social services and national media information systems and, therefore, have better knowledge related to infectious diseases such as TB.

### 4.5 Linkage of knowledge and practice

Overall, good knowledge and good attitudes, as well as poor practices related to TB prevention and control, will guide the methods required to sensitize rural communities, namely including more practical aspects rather than pure transmission of knowledge. We found that participants with higher knowledge have a better attitude and practice in general. Therefore, education campaigns in local areas could be conducted in collaboration with national TB programs in rural communities for food safety hygiene before and after milking and during treatment or birth assistance of animals. This could help communities with good knowledge and attitudes to eventually change their behavior by adopting better practices. It may be of value in future studies to consider barriers to the adoption of good practices. In the current study, we found that, although participants had good attitudes, this did not necessarily translate to good practices; however, we have not yet explored the underlying reason for this observation. Investigations in this direction would be valuable since this can significantly impact the format of education programs. For example, if the principal barrier to the lack of adoption of good practices regarding personal protective equipment is lack of material availability, education programs may need to focus on information regarding the best sources to secure these equipment. Barriers to adoption may also be economic, highlighting the need to integrate free distribution or subsidies for protective equipment. Our study serves as an important foundation for such future work. The African governments are invited to subsidize protective equipment at the national level, and we hope breeders will be able to use them after sensitization and training programs and access them easily in the future.

## 5 Conclusions

This study revealed that persons suspected of having TB in rural Chadian health districts showed good knowledge (90.6%) and attitudes (67.7%) toward TB but had rather poor practices (28.7%). There is, thus, a need for sensitization and practical training and less for providing pure knowledge transfer. However, poor practices may also be due to a lack of accessibility to health centers and means to apply good practices due to the remoteness of the region. It is, therefore, of great importance to improve accessibility to health centers for rural and particularly mobile communities, as well as access to personal protective material. It is equally important to sensitize communities to the zoonotic transmission pathways of TB by promoting food hygiene. Finally, the fact that young people have, throughout the areas studied, been found to show a better overall performance in all three outcome variables (knowledge, attitude, and practices) highlights the potential of involving young people in prevention, surveillance, and control programs in rural health districts. In addition, it also encourages investment in the younger generation to enable behavioral change regarding TB in the future.

## Data availability statement

The original contributions presented in the study are included in the article/Supplementary material, further inquiries can be directed to the corresponding authors.

## Ethics statement

The studies involving humans were approved by the Chadian Bioethics Committee provide Ethical clearance to access to health centers. The studies were conducted in accordance with the local legislation and institutional requirements. The participants provided their written informed consent to participate in this study.

## Author contributions

LD: Writing—original draft, Software, Methodology, Investigation, Formal analysis, Data curation, Conceptualization. MFA: Writing—review & editing, Writing—original draft, Resources, Funding acquisition. NBNR: Writing—review & editing, Writing—original draft, Visualization, Supervision, Project administration, Funding acquisition, Data curation. AD: Writing—review & editing. HK: Writing—review & editing. YI: Writing—review & editing. SDI: Writing—review & editing, Supervision. BAMG: Writing—review & editing, Supervision. JZ: Writing—review & editing, Funding acquisition. BB: Writing—review & editing, Resources, Project administration, Funding acquisition. SDü: Writing—review & editing, Visualization, Validation, Supervision, Software, Funding acquisition, Formal analysis, Data curation.

## Supplementary material

Supplementary Data Sheet 1 Questionnaire used in a study to survey knowledge, attitude, and practices on TB in two rural health districts, Yao and Danamadji, in Chad.

Supplementary Table 1 Database of the responses of the 139 participants of the survey to knowledge, attitude, and practices on TB in two rural health districts, Yao and Danamadji, in Chad.
