# Supplementary material for: Assessment of the knowledge, attitudes, and practices toward human tuberculosis amongst rural communities in Chad
Source: Front Vet Sci. 2024 May 16;11:1334303. doi: 10.3389/fvets.2024.1334303 (PMC11137331; doi:10.3389/fvets.2024.1334303)
Supplement: Supplementary Data Sheet 1 — Questionnaire used in a study to survey knowledge, attitude, and practices on TB in two rural health districts, Yao and Danamadji, in Chad. [file Data_Sheet_1.PDF]

Patient: Name ---

Phone number:______________________________________

Health center of____

Sex: M ☐ F ☐

Age: (year) _____________________

Temperature: ___________

Ethnic group: ____

Profession____

Life style: Settled ☐ Mobile ☐

Clinic:

Causes of the visit at health center**: Treatment** ☐ **Diagnostic** ☐

Specifics signs

- Cough more than two weeks with expectorations : Yes ☐ No ☐
- Thoracic pain : Yes ☐ No ☐
- Hemopstisis : Yes ☐ No ☐
- Presence of ganglion : Yes ☐ No ☐

No specifics signs

- Weight loss : Yes ☐ No ☐
- Tiredness : Yes ☐ Non ☐
- Vesperal fever : Yes ☐ No ☐
- Weakness : Yes ☐ No ☐
- Loss of appetite : Yes ☐ No ☐

- Night sweats : Yes ☐ No ☐

Knowledge: Have you heard about human tuberculosis? Yes ☐ No ☐

and the bovine tuberculosis?:Yes ☐ No ☐

Which channel allow you to hear about the disease? : Radio ☐ Television ☐

Church ☐ Mosque ☐ School ☐ Familial meeting ☐ Job place ☐

Don't know ☐

Is tuberculosis contagious in your ethnic group? Yes ☐ No ☐

Can it be cured? Yes ☐ No ☐

Is it lethal? Yes ☐ No ☐

Do you know the cause of tuberculosis? Yes ☐ Non ☐

Transmission mode: Inhalation ☐ Ingestion ☐ Others ☐

- Is it dangerous? Yes ☐ No ☐
- Is it transmissible between human and animal ?Yes ☐ No ☐

Attitudes :

- Is the sick person excluded from the community?:Yes ☐ No ☐
- Do you think that patients should go to hospital for consultation?: Yes ☐ No ☐
- Do you think that patients should be treated traditionally? Yes ☐ No ☐
- Do you think that patients should take her medication as prescribed? Yes ☐ No ☐

- Do you think that patients should follow the doctor's instructions? Yes ☐ No ☐
- Do you think it is important that patients finish treatment? Yes ☐ No ☐

Pratiques :

- Do you consume raw milk? : Yes ☐ No ☐
- Do you consume boiled milk?: Yes ☐ No ☐
- Do you eat uncooked meat? : Yes ☐ No ☐
- Do you close mouth when you cough? Yes ☐ No ☐
- Do you close windows when you are inside? Yes ☐ No ☐

Specifics questions to breeders :

Do you use protection material during assisting birth for your livestock? (Glove, facial mask)? Yes ☐ No ☐

Do you wash your hands during milking? Yes ☐ No ☐

Do you use protection material during treatment of your animals? (Glove, facial mask): Yes: ☐ No ☐
